# Identification of the Genetic Basis of Phage Resistance in Sequentially Generated Phage-Resistant *Klebsiella pneumoniae* Using an Established Phage Library

**DOI:** 10.3390/antibiotics14111056

**Published:** 2025-10-22

**Authors:** Wenbo Zhao, Congyang Du, Zheng Chen, Yunze Zhao, Stefan Schwarz, Hong Yao, Chenglong Li, Chunyan Xu, Xiang-Dang Du

**Affiliations:** 1 International Joint Research Center of National Animal Immunology, Ministry of Education Key Laboratory for Animal Pathogens and Biosafety, College of Veterinary Medicine, Henan Agricultural University, Zhengzhou 450002, China; zhaowb0101@126.com (W.Z.); 15093286305@163.com (C.D.); zhengchen1214@163.com (Z.C.); 18838180130@163.com (Y.Z.); yaoh0913@henau.edu.cn (H.Y.); mylichenglong@126.com (C.L.); 2 Institute of Microbiology and Epizootics, Centre for Infection Medicine, School of Veterinary Medicine, Freie Universität Berlin, 14195 Berlin, Germany; stefan.schwarz@fu-berlin.de; 3 Veterinary Centre of Resistance Research (TZR), School of Veterinary Medicine, Freie Universität Berlin, 14195 Berlin, Germany

**Keywords:** *Klebsiella pneumonia*, phage resistance, evolutionary trade-off, capsular polysaccharides, phage therapy

## Abstract

***Objectives:*** To explore the genetic basis of phage resistance in sequentially generated capsular mutants of phage-resistant *Klebsiella pneumoniae* using an established phage library. ***Methods:*** Sequential induction strategies were employed to obtain phage-resistant *K. pneumoniae* capsular mutants by exposing ST11-K64 *K. pneumoniae* Kp2325 to different single phages. Whole genome sequencing and bioinformatic analysis were used to elucidate the capsular-related genetic changes in phage-resistant mutants. Phenotypic changes were assessed through gene complementation, growth assays, phage cleavage spectrum analysis, TEM for phage morphology, CPS analysis, biofilm formation, and virulence assays. ***Results:*** Three sequentially generated phage-resistant *K. pneumoniae* capsular mutants were obtained, designated R1, R2 and R3. The narrowing of the phage cleavage spectrum and the evolutionary trade-offs of biological phenotypes were observed. Key genetic changes included: (1) IS*Kpn26* insertion disrupting *wcaJ* in R1; (2) combined *wcaJ* insertion and 9-bp deletion in *waaH* in R2; and (3) CPS gene cluster deletion in R3 were identified as key mechanisms of phage resistance in *K. pneumoniae* mutants R1, R2 and R3, respectively. ***Conclusions:*** Sequential exposure to different single phages led to rapid evolution of phage resistance in *K. pneumoniae* via genetic mutations that disrupt capsular synthesis. These findings highlight the critical role of bacterial capsule in phage–host interactions and emphasize the need to use phage cocktails targeting different types of receptors to counteract the evolution of bacterial defense mechanisms in phage therapy.

## 1. Introduction

*Klebsiella pneumoniae* is a Gram-negative facultative anaerobic bacterium. It can cause infections in multiple body compartments and is a common opportunistic pathogen in clinical practice [1]. It can lead to neonatal sepsis, respiratory and urinary tract infections in immunocompromised individuals, and other multiple-system infections [2]. According to published reports, the number of people infected with *K. pneumoniae* exceeds 90,000 annually, with over 5000 deaths [3]. With the application of carbapenem antibiotics, the occurrence rate of carbapenem-resistant *K. pneumoniae* (CRKP) gradually increased, thereby reducing the number of clinically efficient antibiotics [4]. Therefore, the development of new alternative therapy concepts has become a major public health problem.

Phages are viruses that can infect and lyse bacteria, with the potential to treat severe infections due to drug-resistant bacteria. Not every phage can lyse every bacterium; instead, phages have a strong specificity for their host bacteria. After receptor-mediated attachment to the host bacterium, the lytic phage releases its DNA into the bacterium. The bacterial metabolism is then modified to mainly produce phage particles, which, upon their release, kill the bacterial host cell [5]. Phage therapy is a technique that uses lytic phages to combat bacterial infections, including those caused by multidrug-resistant bacteria [6]. Studies using mice as animal models have shown that phages have good therapeutic effects on pneumonia, liver abscesses, and other infections caused by *K. pneumoniae* [7,8,9]. Multiple studies have shown that phage therapy also has good efficacy in the clinical treatment of urinary tract infections caused by multidrug-resistant *K. pneumoniae* [10,11]. However, the use of phage therapy can easily lead to bacterial resistance, greatly affecting the treatment effect. *K. pneumoniae* strains have developed phage resistance during the course of phage therapy. When bacteria acquire resistance to phages, they often show side effects, such as reduced growth rates and reduced virulence [12]. Although *K. pneumoniae* cannot be eliminated by phages due to resistance, the reduced virulence of phage-resistant strains can still be of benefit to the patients [13]. Therefore, in-depth studies of phage resistance mechanisms play an important role in the prevention and treatment of infections caused by antimicrobial-resistant bacteria.

Phage cocktails are mixtures composed of different phages. They usually have a broader host range than individual phages, and the therapeutic efficacy of various randomized phage mixtures against *K. pneumoniae* has been confirmed by multiple studies [14,15]. When selecting phage cocktail combinations, phages that recognize the same receptors should be avoided because they can act as antagonists to each other [16]. The core of phage cocktail strategies lies in broadening the host range, but their “simultaneous multi-phage pressure” fundamentally differs from the “sequential single-phage pressure” employed in this study: the former requires bacteria to simultaneously counter multiple phage attacks, while the latter involves bacteria gradually adapting to a single phage.

In our study, we subjected a *K. pneumoniae* strain to different phages from an established phage library in our laboratory. Thereby, we obtained several mutant strains that have become resistant to phages targeting different types of receptors. The evolutionary trade-offs of biological phenotypes in phage-resistant *K. pneumoniae* strains were identified and verified. Phage combination preparation targeting different types of receptors in *K. pneumoniae* was formulated based on the phage library to promote the widespread application of phage therapy against *K. pneumoniae*.

## 2. Results

Strategies for the sequential generation of phage-resistant mutant strains.

**Figure 1:**
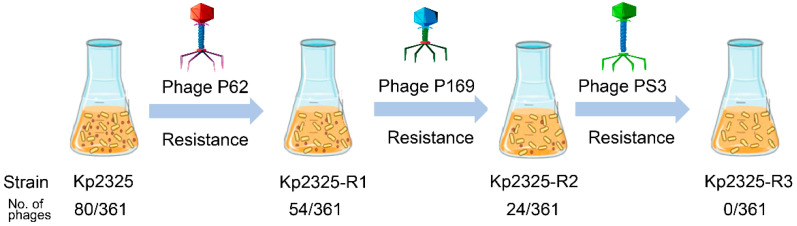
Schematic presentation of the generation of phage-resistant mutant strains. Kp2325 is the original strain. R1 was obtained by co-incubation of phage P62 and Kp2325. R2 was obtained by co-incubation of phage P169 and mutant R1. R3 was obtained by co-incubation of phage PS3 and mutant R2. The phage number indicates the number of effective phages and the total number of phages in the library, respectively.

**Figure 2:**
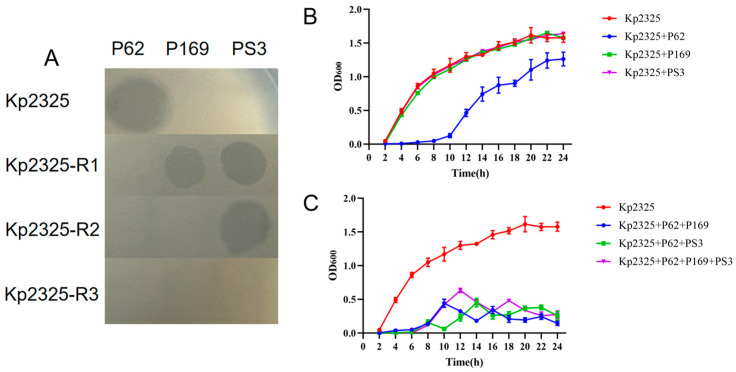
Phage spots and in vitro processing of individual phages and mixtures. (**A**) The phage host spectrums of wild-type and mutant strains used in this study. (**B**) Mixed incubation of a single bacteriophage with the wild strain Kp2325. (**C**) Cocktail and wild strain Kp2325 mixed incubation.

Our laboratory is dedicated to the isolation of phages from effluents from a wide range of environments in order to construct a phage library. To date, 361 non-repetitive phages that can lyse different serotypes of *K. pneumoniae* have been isolated. This phage library will continue to expand with subsequent phage isolations from ongoing sampling. *K. pneumoniae* Kp2325 belongs to the ST11-K64 lineage. Overall, a total of 80 lytic phages were identified in the phage library using Kp2325 as the host bacterium. Phage P62 was randomly selected based on the morphology of the phage spots and co-cultured with Kp2325 to obtain phage resistance. As shown in Figure 1, the first resistant strain mutant was designated Kp2325-R1 (abbreviation for R1), which showed resistance to phage P62. Subsequently, phages from the phage library that could lyse the phage-resistant mutant strain were selected to continue the resistance induction until all phages were disabled. The second resistant strain, Kp2325-R2 (abbreviation for R2), resistant to phages P62 + P169, was obtained using phage P169 induction. The third resistant strain, Kp2325-R3 (abbreviation for R3), resistant to phages P62 + P169 + PS3, was obtained using phage PS3 induction (Figure 1 and Figure 2A). The host range determination results for phages P62, P169, and PS3 are shown in Table 1. The *Klebsiella pneumoniae* strains are used for phage lysis spectrum screening are listed in Table 2.

Screening of phage-resistant mutant strains using a phage library revealed a gradual decrease in the number of effective phages. The original *K. pneumoniae* Kp2325 can be lysed by 80 phages, while R1 and R2 can only be lysed by 54 and 24 phages, and R3 proved to be resistant to lysis by all phages in the phage library. It is worth noting that 83.33% (45/54) of the 54 phages that can lyse R1 were previously ineffective against Kp2325. This observation suggests that *K. pneumoniae* mutants resistant to a specific phage may be susceptible to lysis by other phages than the original parental strain.

### 2.1. Emergence of Phage Resistance and Inhibition of Cocktails

In the case of a single phage, phage P62 effectively inhibited the growth of the original strain Kp2325 within the initial 6 h (Figure 2B). A similar situation occurred in the case of co-incubation using cocktails. All cocktails were effective in inhibiting bacterial growth within 6 h. With the emergence of phage resistance after about 6 h, the resistant mutant strain R1 started to grow. However, the final growth of resistant mutant strains was significantly reduced in the presence of the phage cocktails compared to individual phages, with the most pronounced inhibitory effect observed in the mixture containing phages P62 and P69 (Figure 2C).

### 2.2. Phenotypic Changes in Phage-Resistant Mutants

**Figure 3:**
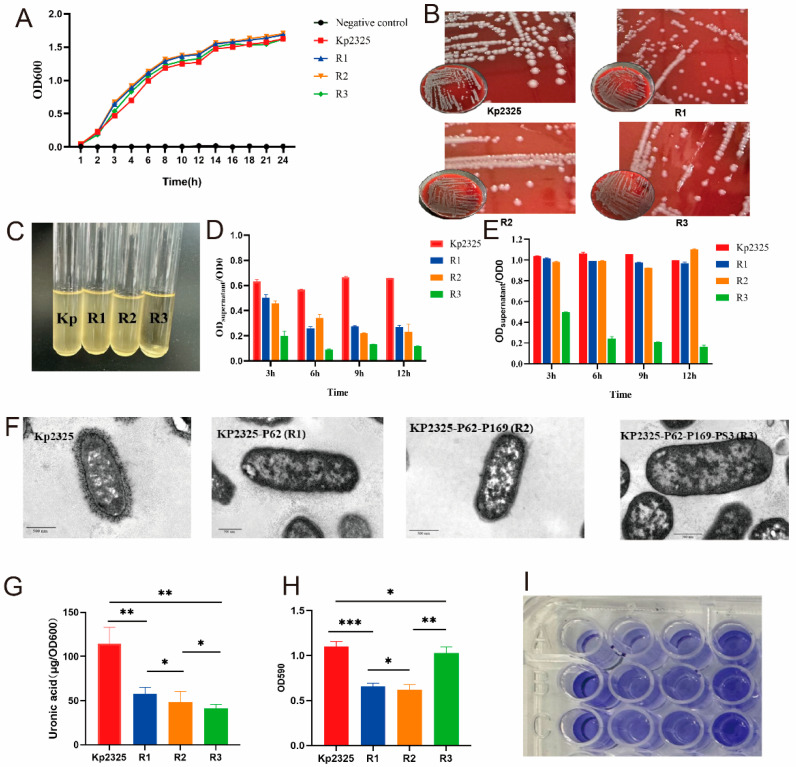
Characterization of phage-resistant mutant strains in comparison to the original strain Kp2325. (**A**) 24 h growth curve measured at 600 nm wavelength. (**B**) The morphology of bacterial colonies grown on a blood plate for 24 h. (**C**) After cultivation in liquid media to the logarithmic growth stage and standing at room temperature for 1 h, the resistant mutant strain R3 had settled. (**D**) The percentage of bacteria that did not settle after the culture was allowed to stand for 1 h following 3, 6, 9, and 12 h of incubation. (**E**) The percentage of bacteria that did not settle after centrifuging the culture at 1000× *g* for 1 min at 3, 6, 9, and 12 h of cultivation. (**F**) TEM images of bacterial cell morphology of Kp2325, R1, R2, and R3. (**G**) The extraction of uronic acid has been carried out for the quantification of the capsule. (**H**,**I**) Crystal violet staining was used to quantify biofilm formation. Absorbance was measured at 590 nm. *, **, and *** indicate significant differences at *P* < 0.05, *P* < 0.01, and *P* < 0.001, respectively.

Acquisition of phage resistance did not affect the ability of bacteria to grow. We observed the 24 h growth of the original strain and the phage-resistant mutant and noticed that Kp2325 had essentially the same growth rate compared to the phage-resistant mutants R1, R2, and R3 (Figure 3A).

Reduced mucus viscosity was observed in the mutant strains. The colony morphology of the phage-resistant mutant strains showed comparatively dry and flattened colonies, whereas the original strain Kp2325 showed moist, prominent, and mucoid colonies (Figure 3B).

After 12 h of incubation in liquid medium and subsequent storage for 1 h at room temperature, the phage-resistant mutant strain R3 sedimented to the bottom of the tube, whereas the medium of the original strain Kp2325 and the mutant strains R1 and R2 remained turbid (Figure 3C). The effect of phage resistance on CPS production was tested by the centrifugal sedimentation test. The results showed that in bacterial suspensions incubated for 3, 6, 9, and 12 h, after brief centrifugation, the original strain had more suspended bacteria than the phage-resistant mutants (Figure 3D). In contrast, in the incubation group that was left to stand for 1 h at room temperature, only the mutant R3 had considerably fewer suspended bacteria (~80% reduction) (Figure 3E). TEM showed a substantial reduction in the thickness of the extra-membranous capsule of *K. pneumoniae* of the mutant strains R1, R2, and R3 compared to the original Kp2325 (Figure 3F). Capsule quantification experiments showed results consistent with TEM observations, i.e., starting from R1, the glyoxylate content was considerably reduced until R3 (Figure 3G). The biofilm measurements showed that the biofilm content of R1 and R2 was lower than that of the original strain, whereas the biofilm formation ability of R3 returned to the level of the original strain (Figure 3H,I).

### 2.3. Quantitation of Phage Adsorption

**Figure 4:**
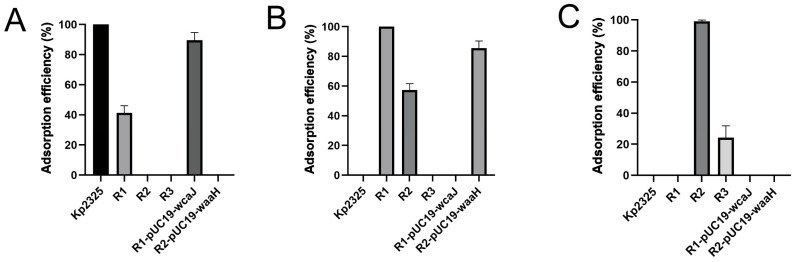
Phage adsorption rate test. Compared with the respective ancestor strain, the adsorption rates of the following resistant mutants decreased, while the complementation strains partially recovered their levels. (**A**) Bacterial Kp2325, resistant mutant R1, complementation strain R1-pUC19-*wcaJ* and phage P62. (**B**) Resistant mutant R1, resistant mutant R2, complementation strain R2-pUC19-*waaH* and phage P169. (**C**) Resistant mutant R2, resistant mutant R3 and phage PS3.

Bacterial mutations that result in altered phage adsorption are one of the most common mechanisms of phage resistance. Quantitative results of phage adsorption can be obtained by measuring free phages after a brief co-incubation. The results showed that R1, R2 and R3 all exhibit a decrease in adsorption efficiency after incubation with phages that have evolved this resistance. In particular, R1 and R3 showed adsorption efficiencies of only 41.2% (Figure 4A) and 24.2% (Figure 4C) compared to the respective direct ancestor strain, while R2 showed 57.3% adsorption efficiency (Figure 4B). However, Phages P169 and PS3 have lost their lytic activity against the Kp2325 strain (Figure 4B,C).

### 2.4. Mechanism of Phage Resistance Mutation

**Figure 5:**
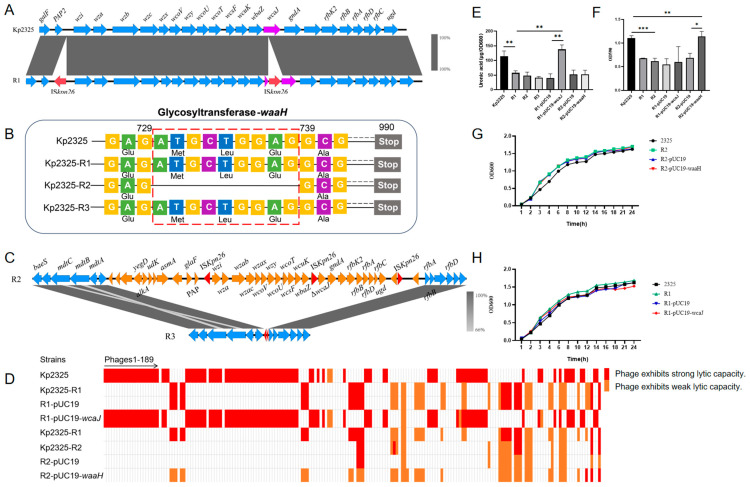
Analysis of the phage resistance factors. (**A**) Schematic diagram of the insertion of IS*Kp26* into the *wcaJ* gene. Arrows of different colors represent different genes, and the direction of the arrows indicates the direction of transcription. (**B**) Schematic diagram of a 9 bp deletion in the *waaH* gene. (**C**) Schematic representation of the CPS gene cluster deletion. (**D**) The range of phage activity on different bacterial strains. Red boxes represent strong lytic activity with clear plaques, while yellow boxes indicate weaker activity with less clear plaques. (**E**) The extraction of uronic acid has been carried out for the quantification of the capsule. (**F**) Crystal violet staining was used to quantify biofilm formation. Absorbance was measured at 590 nm. (**G**,**H**) Determination of the growth curves of the *wcaJ* complemented strain (**G**) and the *waaH* complemented strain (**H**). *, **, and *** indicate significant differences at *P* < 0.05, *P* < 0.01, and *P* < 0.001, respectively.

In order to elucidate the mechanism of successive changes in phage resistance, we sequenced and compared the whole genome sequences of the original strain and the phage-resistant mutant strains. Comparison of the results showed that, in comparison to Kp2325, an insertion sequence of the type IS*Kpn26* was inserted in the *wcaJ* gene in the first resistant mutant R1 (Figure 5A), whereas an additional 9 bp deletion in the *waaH* gene was observed in the second resistant mutant R2 (Figure 5B). In the third resistant mutant R3, there was a large deletion of 50,779 bp in length, comprising a total of 39 CDSs, in the CPS gene cluster (Figure 5C).

To demonstrate that the identified mutations were responsible for the phage-resistance phenotype, the cloned *wcaJ* and *waaH* genes were separately introduced into the R1 and R2 mutants, designated R1-pUC19-*wcaJ* and R2-pUC19-*waaH*, respectively. Further measurements of the susceptibility of R1-pUC19-*wcaJ* and R2-pUC19-*waaH* to phage libraries showed that the complementation of the altered *wcaJ* and *waaH* genes by the corresponding cloned complete genes restored the phage-susceptibility of the mutant strains R1 and R2 (Figure 5D). Interestingly, R1-pUC19*-wcaJ* restored the capsular phenotype of the original strain (Figure 5E), whereas R2-pUC19-*waaH* restored the ability to form biofilms (Figure 5F). We also measured the growth ability of the complemented strains and showed that the pUC19 plasmid carrying *wcaJ* and *waaH* does not have an effect on the growth rate of the host bacteria (Figure 5G,H).

### 2.5. The Polymorphism of waaH Gene Mutations

*WaaH* belongs to the glycosyltransferase 2 family. It encodes a novel glucosyltransferase that is associated with biofilm formation in *E. coli* and is regulated by the transcriptional regulator PhoB [17]. In this study, the *waaH* gene was found to be associated with phage resistance for the first time. To determine the mutational diversity of the *waaH* gene, we expanded the sample size of phage-resistant bacteria and performed sequencing. Two different serotypes of *K. pneumoniae* (Kp2322-ST412-K57 and Kp2324-ST2667-K62) were co-cultured in the presence of phage P169 and five phage-resistant strains were selected for each strain for identification of the *waaH* gene to determine whether P169 stably caused mutations in the *waaH* gene and the direction of the mutations. The results showed that among the 10 phage-resistant mutant strains generated using Kp2322-ST412-K57 and Kp2324-ST2667-K62 as parental strains, all mutants mutated glycine at position 144 to aspartic acid, corresponding to the substitution of g by a at position 431 of the nucleotide (Table 3).

The glycosyltransferase WcaJ is the initiating enzyme of colanic acid synthesis and loads the first sugar (glucose-1-P) on the lipid carrier undecaprenyl phosphate. The absence or functional inactivation of this glycosyltransferase results in the absence of colanic acid, which interferes with capsular polysaccharide formation and, thereby, renders a non-mucoid phenotype to the mutant [18]. Based on our results of sequential generation of phage-resistant mutants of *K. pneumoniae*, we identified the inactivation of the gene *wcaJ* in the first resistant mutant R1. Functional inactivation of the glycosyltransferase-encoding *waaH* gene by deletions, insertions or bp exchanges, as detected in the second mutant R2 and other P169-resistant isolates, also results in impaired capsular polysaccharide formation. In addition, the loss of an entire CPS gene cluster, as seen in the third mutant R3, confirmed that the polysaccharide capsule plays an important role as a phage receptor site.

### 2.6. The Stability of Phage Mutants

**Figure 6:**
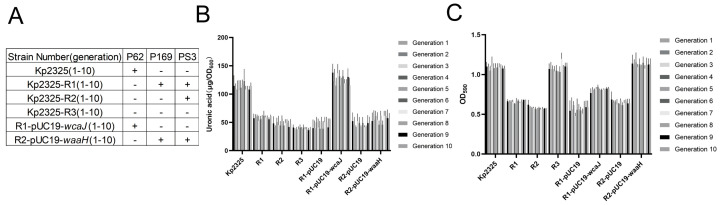
Stability testing of phage-resistant mutant strains. (**A**) Phage susceptibility measured after 10 passages of mutant strains. (**B**) Quantitative analysis of capsular expression in 10-generation strains via aldehydic acid extraction. (**C**) Quantification of biofilm formation using crystal violet staining, with absorbance measured at 590 nm for 10-generation strains.

Phage resistance in these mutants remained stably heritable following 10 consecutive passages. Analysis of phage resistance across 10 successive subcultures confirmed that this resistance phenotype was not lost during the passage process (Figure 6A). Comparative assessment with first-generation phage-resistant mutants revealed that Kp2325, R1, R2, and R3 displayed comparable levels of capsular content and biofilm-forming capacity (Figure 6B,C). Collectively, these results verify that the phage-resistant phenotype in the mutants is stably inherited by progeny strains.

### 2.7. Phage-Resistant Strains Show Reduced Virulence in Mouse Models

**Figure 7:**
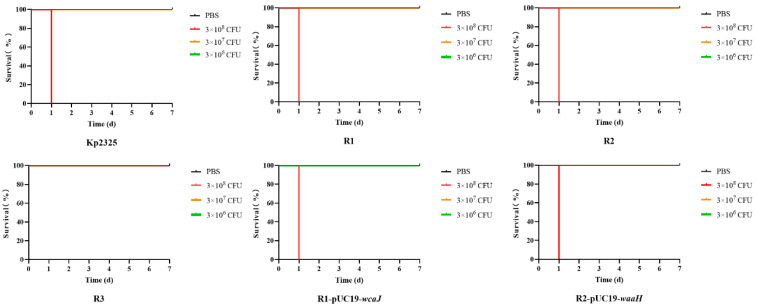
Assessment of the virulence of bacteria by the survival curves of mice. All mice were intraperitoneally injected with a dose of 0.1 mL, with six mice per group. The survival status of the mice was observed and recorded daily for a period of seven days.

The in vivo virulence of the bacteria was further determined by plotting the survival curves of mice infected with each bacterium. All strains were injected intraperitoneally at doses of 3 × 10^8^, 3 × 10^7^ and 3 × 10^6^ CFU/mouse, respectively. According to the 7-day test results, Kp2325, R1, R2, R1-pUC19-*wcaJ*, and R2-pUC19-*waaH* strains injected at a dose of 3 × 10^8^ CFU/mouse caused severe respiratory symptoms in mice within 6 h, and all mice died within one day. All mice injected at a dose of 3 × 10^7^ CFU/mouse survived after a brief period of mild respiratory symptoms, while mice injected with R3 survived even at a dose of 3 × 10^8^ CFU (Figure 7). These results indicate that the virulence of R3 was reduced compared with ST11-K64 *K. pneumoniae* strain Kp2325 and its phage-resistant and complemented mutants (R1, R2, R1-pUC19-*wcaJ*, and R2-pUC19-*waaH*). Even at the highest inoculating dose, R3 did not cause death in mice. This difference between the strains suggested that in the absence of CPS, ST11-K64 *K. pneumoniae* strain Kp2325 revealed reduced virulence.

## 3. Discussion

Carbapenem-resistant high-risk *K. pneumoniae* ST11-K64 strains, such as Kp2325, are able to survive in environments where antibiotics are commonly applied, thereby posing a significant threat to healthcare networks [19]. In this study, we generated Kp2325 mutant strains that were resistant to one, two and all three representative phages from our phage library. The third resistant mutant, R3, could not be lysed by any of the phages in our library. Even attempts to isolate phages from multiple sources of effluents using R3 as the parental strain did not yield effective phages. However, we found that the use of a phage cocktail could exert some growth inhibition on the R3 mutant, although not kill it.

In the interaction between phages and *K. pneumoniae*, phage resistance is gained at the cost of adaptive phenotypic changes [20]. In this study, the colony morphology of the original strain Kp2325 was mucoid, whereas the resistant mutant strains exhibited dry, flat and less mucoid colonies. A significant change in the capsule phenotype of the phage-resistant mutants was confirmed by TEM. Whole genome sequencing and cloning of the genes indicated that the process was due to the disruption of the *wcaJ* gene by IS*Kpn26* insertion in the R1 mutant. The *wcaJ* gene encodes a glycosyltransferase, which is the initiating enzyme of the colanic synthesis pathway, and plays an important role in the synthesis of the capsule [21]. Several studies have reported that the capsule of *K. pneumoniae* plays an important role in the interaction with phages. Mobile genetic elements, such as IS*Kp26*, can lead to phage resistance in *K. pneumoniae* by disrupting the coding region of *wcaJ* [22]. In hypervirulent *K. pneumoniae* (HvKP), mutations in *wcaJ* affect the synthesis of the strain’s capsule, thereby inhibiting phage adsorption [23]. The original strain Kp2325 in this study was lysed by 80 phages when the *wcaJ* gene was functionally active, but only by 54 phages when *wcaJ* was inactivated. Interestingly, 45 of the 54 lytic phages were new phages in addition to the original 80 phages, and the remaining nine phages formed very weak phage spots. The capsule typically masks molecules on the bacterial outer membrane, thus preventing the phage from directly binding to these potential receptors [24]. As a result, many phages have evolved the ability to use the capsule as their primary receptor; they first bind to the capsule and then degrade the capsule, which allows the phages access to their receptors on the cell membrane [25]. Therefore, the capsule not only serves as a target for phage recognition but also as a barrier to prevent other phages from recognizing receptors underneath the capsule.

For the generation of the second phage-resistant strain, we used phage P169 and obtained a resistant mutant in which a 9 bp deletion of the *waaH* gene had occurred. The biofilm-forming ability of the strain was drastically reduced, and the biofilm level was restored after complementation of the mutant with the original *waaH* gene. The *waaH* gene encodes a glycosyltransferase, which is associated with the modification of LPS [17] by catalyzing the addition of glucuronic acid to the third heptose of the inner core oligosaccharide [26]. The *waaH* gene transcription is positively regulated in a growth-phase-dependent manner by a two-component system of PhoB/R [27]. In the present study, the loss of the capsule in *K. pneumoniae* Kp2325 led to changes in the bacterial surface structure. *WaaH*, an enzyme involved in LPS core synthesis, modifies surface components that may serve as secondary binding sites for phage P169. Thus, while *WaaH* itself is not a surface receptor, its activity indirectly affects phage binding. Besides the 9 bp deletion in the *waaH* gene observed in the mutant R2, investigation of other *K. pneumoniae* strains in the presence of phage P169 identified numerous other modifications in the *waaH* gene. Among them, 100% (10/10) of the changes in Kp2322-ST412-K57 and Kp2324-ST2667-K62 resulted in the amino acid (aa) exchange of G144D. This observation might suggest that the glycine residue at position 144 plays an important role (Table 1).

The mutant R2 of *K. pneumoniae* Kp2325 was subjected to exposure to the phage PS3, which resulted in the generation of the third resistant mutant R3. In the R3 mutant, a complete loss of the CPS gene cluster, which was still present in the mutant R2, was observed. Large deletions including the CPS gene cluster have been detected before [28]. Surprisingly, during the generation of the third resistant strain R3, the 9 bp deletion in the *waaH* gene was restored to its original state, and the level of biofilm formation was restored to a certain extent; however, the phage-susceptible phenotype was not restored. Previous studies have shown that disruption of *wcaJ* is a known phage resistance mechanism and is accompanied by a concomitant decrease in virulence [29]. However, in the present study, disruption of *wcaJ* did not lead to a decrease in virulence, whereas loss of the complete CPS cluster did. Therefore, in non-highly virulent CRKP, the contribution of the CPS gene cluster to virulence was greater than that of *wcaJ*. We observed that the loss of the CPS gene cluster in *K. pneumoniae* Kp2325 reduced capsule production, likely diminishing bacterial adherence to host tissues and increasing susceptibility to host immune defenses, particularly phagocytosis. This highlights the critical role of CPS in mediating both virulence and resistance to clearance by the immune system.

## 4. Materials and Methods

### 4.1. Bacterial Strains and Culture Conditions

The CRKP Kp2325 strain, isolated from a clinical sample [30], and the phage library (isolated from pig farm water samples in Henan Province using the double-layer agar method [31]) were used. All strains and phages were stored at −80 °C after purification. Fresh bacterial cultures were grown in LB broth (Hopebio, Qingdao, China) at 37 °C for 16 h with shaking (Huamei Biochemical Instrument Factory, Taicang, China).

### 4.2. Host Range of the Phages

The host range of the phages was determined using the spotting method [32], i.e., 200 μL of fresh bacterial culture was added to 8 mL of 0.6% top agar and poured into the lower LB agar plate. After the agar solidified, 5 μL of phage was added dropwise to the top of the bilayer plate and incubated at 37 °C overnight.

### 4.3. Phage Adsorption

To determine the phage adsorption efficiency on host bacteria, the procedure described previously was followed with appropriate modifications [33]. Host bacteria were cultured to the logarithmic growth phase, phage was added at an MOI of 0.1, and co-incubation was performed at 37 °C and 180 rpm. After 10 min, incubation was terminated. The mixture was centrifuged at 12,000× *g* for 2 min, filtered through a sterile 0.22 μm membrane (Millipore, Billerica, MA, USA), and 100 μL of the filtrate was subjected to tenfold serial dilutions in sterile LB broth. to determine the phage titer. Simultaneously, the phage added at the start of the experiment was also subjected to a tenfold serial dilution and counted using the double-layer plate method to establish the initial phage amount. Observe results after 8–12 h, count, and plot adsorption efficiency. Adsorption efficiency = [(Initial phage amount − Titration in supernatant)/Initial phage amount] × 100%.

### 4.4. Phage Resistance Suppression Test

Three individual phages (P62, P169, PS3) and four phage cocktails (P62 + P169, P62 + PS3, P169 + PS3, P62 + P169 + PS3) were prepared. Bacterial cultures were incubated with phages (MOI = 0.1) at 37 °C for 24 h, with OD600 measured every 2 h using a Multiskan SkyHigh Microplate Spectrophotometer (Thermo Fisher Scientific, Waltham, MA, USA). All tests were repeated three times.

### 4.5. Genome Sequencing and Analysis

The whole genome DNA of CRKP Kp2325, R1, R2 and R3 were extracted using the TianGen DNA Extraction kit (TianGen, Beijing, China) following the manufacturer’s instructions. The 250 bp paired-end reads were obtained using an Illumina MiSeq system (Illumina, San Diego, CA, USA). Draft genomes were assembled using SPAdes. MinION long-read sequencing was then performed to obtain the complete genome sequence of the strains. The complete genome sequence was annotated using RAST (https://rast.nmpdr.org/rast.cgi (accessed on 1 August 2025)). Comparisons of the genetic environment were illustrated using Easyfig 2.2.3.

### 4.6. Cloning Experiments to Investigate Phage Resistance Mechanisms

Cloning experiments confirmed that disruptions in the *wcaJ* and *waaH* genes mediate phage resistance in *K. pneumoniae*. These genes were amplified using self-designed primers via reverse PCR and cloned into the pUC19 plasmid using the Trelief^®^ Seamless Cloning Kit (Tsingke, Beijing, China). The PCR primers used for cloning are listed in Table S1. The resulting recombinant plasmids (pUC19-*wcaJ* and pUC19-*waaH*) were initially transformed into *Escherichia coli* DH5α and selected on apramycin-containing (100 μg/mL) LB agar plates. For electrotransformation into recipient *K. pneumoniae* strains R1 or R2, electrocompetent cells were prepared by culturing in LB medium at 37 °C with shaking at 200 rpm until OD_600_ reached 0.5. Cultures were incubated on ice for 30 min, centrifuged at 4 °C and 5000× *g* for 10 min, and washed three times with pre-chilled sterile 10% glycerol under the same centrifugation conditions. The final cell pellet was resuspended in 100 μL of pre-chilled 10% glycerol and stored at −80 °C until use. For electrotransformation, 100 ng of recombinant plasmid was mixed with 50 μL of competent cells and transferred to a pre-chilled 0.1 cm electroporation cuvette (Bio-Rad, Cat. No. 1652086). Electroporation was performed using a GenePulser Xcell system (Bio-Rad) under the following conditions: 2.5 kV, 25 μF, 200 Ω. Immediately after electroporation, 1 mL of LB broth was added, and cells were incubated at 37 °C with shaking at 200 rpm for 1 h. A 100 μL aliquot was plated on LB agar containing 100 μg/mL apramycin and incubated at 37 °C for 16 h. Single colonies were selected and verified by PCR using insert-specific primers. Successful gene cloning and restoration of phage susceptibility were confirmed by PCR and spot assay experiments.

### 4.7. Preparation and Quantification of Capsules

The quantification of capsules was performed using colorimetric extraction of glucuronic acid content [34]. Bacterial cultures at the stationary phase were mixed with 1% zwittergent 3-14 (Macklin Biochemical, Shanghai, China) in 100 mM citric acid (pH 2.0) (Macklin Biochemical, Shanghai, China) and incubated at 50 °C for 30 min. After centrifugation at 12,000× *g* for 2 min, the supernatant was treated with anhydrous ethanol (Macklin Biochemical, Shanghai, China), followed by centrifugation and resuspension in ddH_2_O. The mixture was then boiled with borax in H_2_SO_4_ (Beijing Solarbio Science & Technology Co., Ltd., Beijing, China) and cooled with 3-hydroxydiphenol in NaOH (Beijing Solarbio Science & Technology Co., Ltd., Beijing, China). Absorbance was measured at 490 nm using a Multiskan SkyHigh (Thermo Fisher Scientific, Shanghai, China).

### 4.8. Biofilm Formation

A 200 µL bacterial suspension was diluted in LB broth and dispensed into 96-well plates and incubated at 37 °C for 48 h. The plates were washed with PBS, fixed in methanol, stained with 0.1% crystal violet, and eluted with 30% glacial acetic acid. Absorbance was measured at 590 nm using UV spectrophotometry (U-3900/3900H, Hitachi High-Tech, Tokyo, Japan). Each experiment was replicated three times.

### 4.9. Mutation Analysis of the waaH Gene

To determine whether phage P169 induces stable mutations in the *waaH* gene of *K. pneumoniae*, two serotypes (Kp2322-ST412-K57 and Kp2324-ST2667-K62) were co-cultured with phage P169 (MOI = 0.1) in 100 mL LB medium at 37 °C with shaking at 200 rpm (Huamei Biochemical Instrument Factory, Taicang, China). Samples were taken periodically, and the *waaH* gene was sequenced to identify mutations. The direction of mutation was determined by comparing the mutated sequence with the wild-type sequence.

### 4.10. Stability of Phage Mutants

R1, R2, and R3 strains and other phage mutants were separately inoculated into phage-free LB broth and incubated at 37 °C with constant shaking at 200 rpm. Every 24 h, the cultures were subcultured into fresh LB broth at a 1:100 dilution ratio, and this subculturing process was continued for 10 consecutive passages. The sensitivity of the strains to phages P62, P169, and PS3 was assessed using the spot test method, as described in Section 2.2. For the 10th-passage phage-resistant mutants, capsular polysaccharide (CPS) content and biofilm-forming ability were determined via the glucuronic acid colorimetric assay and crystal violet staining method, respectively.

### 4.11. Bacterial Virulence Assay in Mouse Infection Model

All animal experiments and protocols were conducted in accordance with the guidelines approved by Henan Agricultural University Scientific Ethics Committee (approval numbers HNND2025071401). Six-week-old female BALB/c mice were obtained from the Huaxing Animal Farm in Huiji District, Zhengzhou, China. The mice were housed under controlled conditions at a temperature of 22 ± 2 °C with a 12 h light–dark cycle. They had ad libitum access to food and water and were acclimated to these conditions for one week prior to the start of the experiments.

Six-week-old BALB/c mice were used, with six mice per bacterial dose group (*n* = 18). Each infecting strain was cultured to the logarithmic growth stage, and approximately 3 × 10^8^ CFU were diluted in PBS. Mice were infected intraperitoneally with 0.1 mL of the bacterial suspension, with PBS as a negative control. Survival rates were recorded daily for seven days. The mice in all groups were housed under identical conditions, and the investigator assessing the various parameters was blinded to the group allocations. At predetermined time points, mice were euthanized Via cervical dislocation.

### 4.12. Statistical Analysis

Data were analyzed using two-tailed Student’s *t*-tests in GraphPad Prism 9 (GraphPad Software Inc., San Diego, CA, USA), and results are presented as mean ± standard deviation (SD). Statistical significance between groups was determined by the *t*-test, with *p*-values less than 0.05 considered statistically significant.

## 5. Conclusions

This study elucidates the dynamic process of *K. pneumoniae* in developing resistance to phages, a phenomenon that poses significant challenges to the efficacy of phage therapy. Through a systematic approach of generating and characterizing phage-resistant mutants, we have identified key genetic alterations that explain the resistance mechanisms. The narrowing of the phage cleavage spectrum and changes in the capsule were observed as the primary phenotypic changes in resistant strains. Whole genome sequencing revealed a sequential development of resistance, pinpointing the genes *wcaJ* and *waaH*, as well as the CPS gene cluster, as critical factors. These findings underscore the complexity of phage–host interactions and the need for a multifaceted strategy in phage therapy.

## Figures and Tables

**Table 1:** Host Range Determination Results for Bacteriophages P62, P169, and PS3.

Phage Number	Phage Plaque Morphology	Host Bacterium Serotype	Cleavable Serotype
P62	Medium-Translucent	K64	3 (K3, K15, K64)
P169	Small-Translucent	K2	7 (K2, K15, K19, K57, K62, K70, K74)
PS3	Medium-Translucent	K2	3 (K2, K38, K57)

**Table 2:** *Klebsiella pneumoniae* is used for phage lysis spectrum screening.

Strain	MLST Type	K Type	Number of Effective Phages
KP2301	ST23	K1	17
KP2302	ST86	K2	16
KP2303	ST395	K3	105
KP2304	ST1049	K5	0
KP2305	ST22	K9	2
KP2306	ST3406	K10	0
KP2307	ST37	K12	1
KP2308	-	K14	1
KP2309	ST477	K15	103
KP2310	ST963	K16	3
KP2311	ST1	K19	36
KP2312	ST3355	K20	2
KP2313	ST15	K24	0
KP2314	ST11	K25	1
KP2315	ST15	K28	24
KP2316	ST342	K30	1
KP2317	ST37	K38	8
KP2318	ST4860	K39	1
KP2319	ST11	K47	25
KP2320	-	K48	1
KP2321	ST29	K54	0
KP2322	ST412	K57	15
KP2323	-	K60	0
KP2324	ST2667	K62	7
Kp2325	ST11	K64	112
KP2326	-	K70	1
KP2327	ST273	K74	9

**Table 3:** Identification of *WaaH* amino acid polymorphisms.

Parental Strain	Resistant Strain	Mutations in *WaaH*
Kp2322-ST412-K57	Kp2322-1	G144D, L301V
	Kp2322-2	G144D
	Kp2322-3	G144D
	Kp2322-4	G144D
	Kp2322-5	G144D
Kp2324-ST2667-K62	Kp2324-1	G144D
	Kp2324-2	G144D, T230S, G272W
	Kp2324-3	G144D
	Kp2324-4	G144D
	Kp2324-5	G144D, H223Y, R236L

Note: Kp2322-ST412-K57 and Kp2324-ST2667-K62 were used as parental strains, and P169 was used to induce the production of phage resistance. Five phage-resistant strains were selected for each parent strain, designated 1–5, respectively.

## Data Availability

The sequence assemblies of the CRKP Kp2325 strain and phages have been deposited in the NCBI database under accession numbers PRJNA1229512.

## Supplementary Materials

The following supporting information can be downloaded at: https://www.mdpi.com/article/10.3390/antibiotics14111056/s1, Table S1: PCR primers used for cloning.
