# Supplementary material for: Identification of the Genetic Basis of Phage Resistance in Sequentially Generated Phage-Resistant Klebsiella pneumoniae Using an Established Phage Library
Source: Antibiotics (Basel). 2025 Oct 22;14(11):1056. doi: 10.3390/antibiotics14111056 (PMC12649466; doi:10.3390/antibiotics14111056)
Supplement: Supplementary file 1 [file antibiotics-14-01056-s001.zip › antibiotics-3845101-supplementary.pdf]

Supplementary Material

Table S1 PCR primers used for cloning.

Primer	Sequence (5'-3') *	Target gene	Amplicon size (bp)
pUC19-res-F	CTGTCAGACCAAGTTTACTCATATATACTTT	pUC19	1,825
pUC19-res-R	ACTCTTCCTTTTTCAATATTATTGAAGC		
aac(3)-IVa-F	GCTTCAATAATATTGAAAAAGGAAGAGTGATGTTATGGAGCAGCAACGAT	*aac*(3)-IVa	844
aac(3)-IVa-R	TCTAAAGTATATATGAGTAAACTTGGTCTGACAGATCTCGGCTTGAACGAATTGTCAG		
pUC19-AP-F	TCGAATTCACTGGCCGTCGT	pUC19-AP	2,657
pUC19-AP-R	GGGATCCTCTAGAGTCGACCTGCA		
wcaJ-F	GCAGGTCGACTCTAGAGGATCCCATGAAAACTTTCACGCATCG	*wcaJ*	1,398
wcaJ-R	ACGACGGCCAGTGAATTCGATCAATATGCAGACTTATTAATAAACCCT		
waaH-F	GCAGGTCGACTCTAGAGGATCCCATGAGTCAAACGCCTTTATTG	*waaH*	990
waaH-R	ACGACGGCCAGTGAATTCGACTATCCCGCGTGGCGATAA		

*underlined ends indicate homology arms
